# Sensitive impedimetric biosensor for direct detection of diazinon based on lipases

**DOI:** 10.3389/fchem.2014.00044

**Published:** 2014-07-04

**Authors:** Nedjla Zehani, Sergei V. Dzyadevych, Rochdi Kherrat, Nicole J. Jaffrezic-Renault

**Affiliations:** ^1^UMR 5280, Institut des Sciences Analytiques, Université de LyonVilleurbanne, France; ^2^Laboratory of Environmental Engineering, Faculty of Engineering Sciences, University of AnnabaAnnaba, Algeria; ^3^Laboratory of Biomolecular Electronics, Institute of Molecular Biology and Genetics, National Academy of Sciences of UkraineKiev, Ukraine

**Keywords:** biosensor, diazinon, lipase, electrochemical impedance spectroscopy

## Abstract

Two novel impedimetric biosensors for highly sensitive and rapid quantitative detection of diazinon in aqueous medium were developed using two types of lipase, from *Candida Rugosa* (microbial source) (CRL) and from porcine pancreas (animal source) (PPL) immobilized on functionalized gold electrode. Lipase is characterized to specifically catalyze the hydrolysis of ester functions leading to the transformation of diazinon into diethyl phosphorothioic acid (DETP) and 2-isopropyl-4-methyl-6-hydroxypyrimidine (IMHP). The developed biosensors both presented a wide range of linearity up to 50 μM with a detection limit of 10 nM for *Candida Rugosa* biosensor and 0.1 μM for porcine pancreas biosensor. A comparative study was carried out between the two biosensors and results showed higher efficiency of *Candida Rugosa* sensor. Moreover, it presented good accuracy and reproducibility, had very good storage and multiple use stability for 25 days when stored at 4°C.

## Introduction

Chemicals released from agriculture or industry may potentially develop toxic effects in the environment and ecological systems. Among them, pesticides are actively applied and globally used for crop control and to prevent damage to plants, animals, humans, and aliments. Organophosphates constitute the most extensive and manifold group of pesticides, they were developed at the beginning of this century by chemical manipulation of nerve gasses that are so toxic compounds (Osterauer and Kohler, 2008). Their mode of toxicity is the inhibition of acetyl cholinesterase, an enzyme responsible for the hydrolysis of the neurotransmitter acetylcholine (Cabello et al., 2001; Gordon and Mack, 2003; Pesando et al., 2003). This inhibition leads to a continuous stimulation of cholinergic neurons and eventually paralyzes the target organs (Wiener and Hoffman, 2004).

Diazinon after malathion is one of the most commonly used organophosphate pesticides (OPs) in the world, it is extensively used as an insecticide in agriculture to control juvenile forms of insects in soil, plants, fruit, vegetable crops and to control external pet parasites (i.e., mites, leaf miner flies, black cherry aphid, and apple maggot) (Karpouzas and Singh, 2006; UAP. Ca, 2007), it can enters the body via skin contact, feeding, and inhalation (Villeneuve et al., 1972). Furthermore, diazinon is one of the substances most responsible for acute poising via insecticides of humans and wildlife (Keizer et al., 1995). Once applied on crops and other plants, it is easily washed by surface waters and enters the ground water. Eventually, it enters the aquatic environment in large quantities as described in a number of studies and thus may affect a wide range of non-target organisms. The high acute toxicity of diazinon to freshwater fish and aquatic invertebrates is reflected by the 96 h LC50 of 2 g/L in *Daphnia magna*, of 1.35 mg/L in *O. mykiss*, and of 8 mg/L in *Danio rerio* (Osterauer and Kohler, 2008). Therefore, the persistence and mobility of diazinon and its metabolites suggest the potential for groundwater contamination, an increasing concentration of diazinon and OPs residues are found to be present in many sampled soils, aquatic eco collected in the United States and Canada and it was the most frequently detected insecticide in surface waters prior to the phase-out of urban uses in 2004 in United States. It degrades in water as a result of hydrolysis, especially under acidic conditions. In sterile water, diazinon was determined to have a half-life of 12 days in acidic water (pH 5), 138 days in neutral water (pH 7), and 77 days (pH 9). Moreover, because diazinon is fat soluble, there is potential for delayed toxicity if significant amounts of diazinon are stored in fatty tissues and causes diseases with long-term (US NPIC, 2009). Diazinon applied to soils can be also absorbed by plant roots and translocated in plants, soil metabolism studies report soil half-lives for diazinon ranging from 21 to 103 days depending on the type of soil. In addition, oxypyrimidine which is the principal metabolite of diazinon hydrolysis is very mobile in the environment and has been measured up to 72 inches below the surface of soils. Oxypyrimidine appears to be more persistent under at least some conditions compared with diazinon (US NPIC, 2009).

Thus, the need to be understood and evaluate the biological effects of pollutants on aquatic ecosystems has generated (Reddy et al., 2013). In this sense, a large number of studies have used biosensors as functional tools to evaluate the toxicity of such compounds for natural populations (Kumar and D'Souza, 2011). Recently, there has been an intense research effort to develop enzymatic biosensor devices for the detection of organophosphorus pesticides. Many enzymes were used for this purpose (Pogacnik and Franco, 1999; Deo et al., 2005; Sajjadi et al., 2009). Among them, we find the lipase which it is an important enzyme in biological systems, where is catalyzes the hydrolysis of ester functions and the transformation of triacylglycerol to glycerol and fatty acids, it is a subclasses of esterases (Bhalchandra et al., 2008).

**Figure 1:**
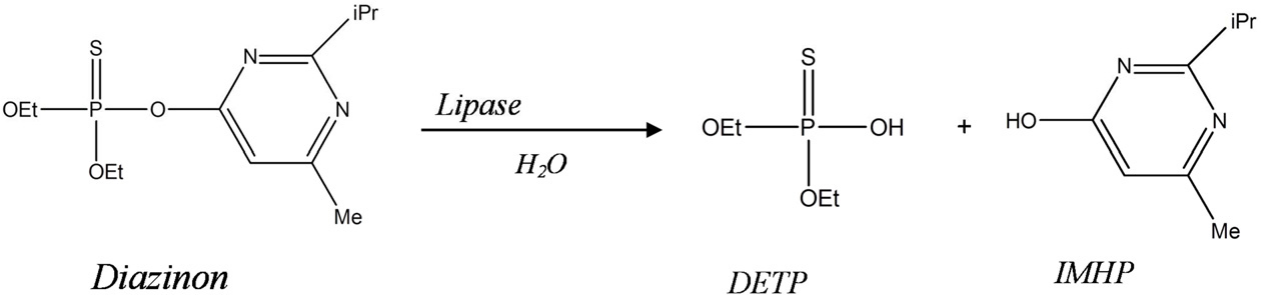
**Degradation pathway of diazinon by lipase (Hydrolysis reaction)**.

This paper describes a new biosensor system devoted for environmental application, based on impedimetric transduction incorporating two types of enzymes, lipase from *Candida Rugosa* CRL (microbial source) and lipase from porcine pancreas PPL (animal source) for the detection of diazinon in aqueous medium. This is the first time that lipase is proposed for the design of diazinon biosensor. This enzyme was used to catalyze the hydrolysis of diazinon (O,O-diethylO-(2-isopropyl-6methyl-4-pyrimidyl phosphorothioate) into Diethyl phosphorothioic acid (DETP) and 2-isopropyl-4methyl-6 hydroxypyrimidine (IMHP) (Figure 1). In this work, bioselective membranes were prepared by functionalization of microelectrodes with SAMs and enzyme cross-linking using glutaraldehyde vapor and bovine serum albumin (BSA). As a detection mode, we used electrochemical impedance spectroscopy (EIS) which is one of the electrochemical techniques that have been widely employed to study various chemical and biological phenomena on surfaces and to develop sensors (Yagati et al., 2011). EIS has emerged as a powerful tool to study the biomolecular interactions by detecting changes in capacitance and interfacial electron transfer resistance at the surface electrode occurring during these processes (K'Owino and Sadik, 2005; Ferreira et al., 2010). It is a rapidly developing technique for the label free detection of different types of biosensing events occurring at the surface electrode, example: antigen-antibody enzyme-substrate reaction, cell adsorption (Bourigua et al., 2010), it allows such complex recognition events to be probed in a simple, sensitive, label-free, and mediator free strategy (Sadik et al., 2002; Farcas et al., 2010). In this study, the different functionalization steps of gold microelectrodes were first characterized by EIS and cyclic voltammetry (CV), afterward, the analytical characteristics of the developed biosensors were determined. Finally, a comparison study between the two biosensors was carried out.

## Experimental

### Chemical and biological reagents

All reagents used in this study were purchased from Sigma Aldrich (Saint Quentin Fallavier) France, including, lipase from *Candida Rugosa* enzyme (CRL, type VII, ≥700 unit/mg solid), Lipase from porcine pancreas (PPL, type II, 30–90 units/mg protein), bovine serum albumin (BSA), diazinon, parathion methyl, paraoxon methyl, atrazine, sevin, simazine, fenitrothion, sodium phosphate dibasic, sodium phosphate monobasic, KH_2_PO_4_, K_2_HPO_4_, glutaraldehyde (grade II, 25% aqueous solution), glycerol (≥99%), 6-methyl-5propyl-4 pyrimidinone, N-hydroxysuccinimide (NHS), 1-ethyl-3(3-(dimethyl-amino)propyl)carbodiimide (EDC), acidthiol(16mercacaptohexadecanoicacid). Sulfuric acid (96%), hydrogen peroxide (30%), ethanol (99%) was purchased from Fluka. All solutions were made up with ultrapure water (resistivity no less than 18 MΩ cm and obtained from a Millipore purification system).

### Electrode gold preparation and enzyme immobilization

#### Gold cleaning

Before analysis, in order to improve the adhesion of the enzymatic membrane on the electrode surface, gold electrode was firstly immersed in acetone for 10 min under sonication to remove the resin layer; then, it was immerged for 3 min in Piranha solution (H_2_O_2_:H_2_SO_4_: 3:7 v/v). Finally the electrode was thoroughly rinsed with ultrapure water and dried under a nitrogen flow.

#### Functionalization and activation process

The gold electrode was incubated overnight in 10 mM solution of thiolacid in ethanol, overnight at a temperature of 4°C, which allowed the formation of a self-assembled monolayer (SAMs) on the surface of the electrode. The thiol functionalized electrode was then rinsed with ethanol in order to remove the unbonded thiol molecules. The resulting monolayer ending with carboxylic acid groups were then activated by an [EDC/NHS] mixture at a concentration of 0.1 mol·l^−1^ for 1 h.

#### Enzyme attachment

Afterward, the pretreated electrode was rinsed with PBS and dried under nitrogen flow, 10 μL of enzyme based solution containing CRL (5%), BSA (5%), glycerol (10%) in phosphate buffer solution 20 mM, pH = 7.2 (90%) was thoroughly homogenized and deposited onto the surface of the working electrode. Then, the sensor was placed in saturated glutaraldehyde vapor (cross-linker) for 30 min, and dried in air at room temperature for 40 min, the biosensor was stored at 4°C until further use.

### Electrochemical set-up: cyclic voltammetry (CV) and electrochemical impedance spectroscopy (EIS)

Electrochemical measurements were carried out in a conventional one compartment three electrode cell with an internal volume of 5 ml (Verre equipments Collonges au Mont d'or, France), hermetically closed on one side with a planar gold electrode (300 nm thickness, deposited on insulated silicon with 0.19 cm^2^ surface area) used as the working electrode. On the other side, a planar platinum electrode (0.59 cm^2^) was used as the counter electrode. A saturated calomel electrode from Hach Lange (France) was used as reference electrode. This electrochemical cell was designed to maintain a fixed distance between the electrodes. It was manufactured with two inlets; one for the positioning of the reference electrode and the other for OP injections. This feature prevented further manipulation or movements of the electrodes, fixing the geometry of the cell and also ensuring the reproducibility of measurements.

**Figure 2:**
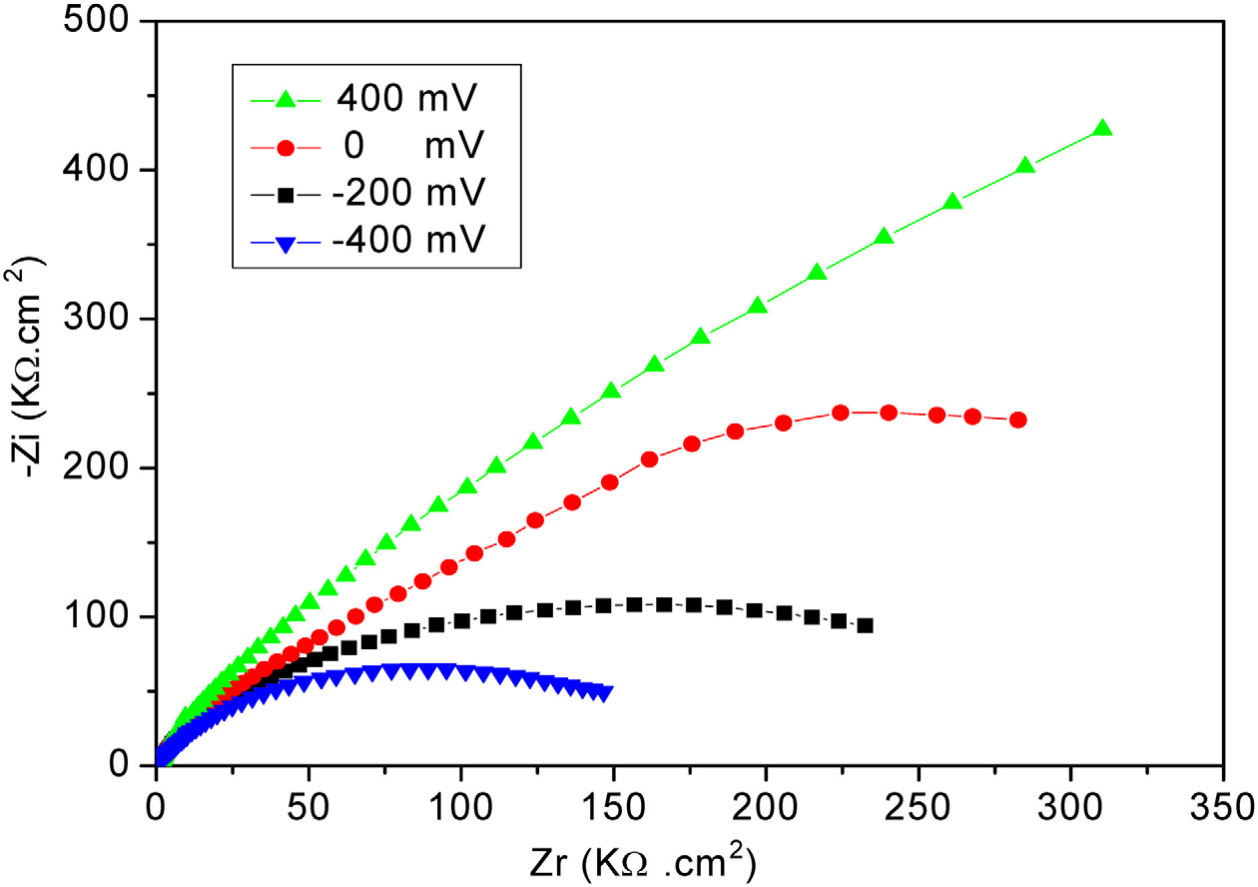
**Nyquist plots obtained for the developed biosensor at different applied potentials**. Measurements were performed in PBS 20 mM, pH 5.2, for frequencies ranging from 100 mHz to 100 kHz.

**Figure 3:**
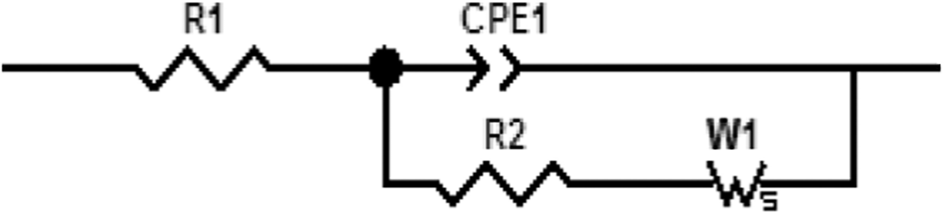
**Randles equivalent circuit**.

The electrochemical measurements were performed with an electrochemical impedance analyzer “Voltalab PGZ 402” (Hach Lange, France). Data acquisition and processing via “Voltamaster 4” software was provided by Thesame company. CV measurements were performed in 8 mM solution of [Fe (CN_6_)]^3−/4−^ at scan rate of 100 mV/s. All electrochemical measurements were taken at a frequency range of 100 mHz–100 kHz at room temperature. Measurements were performed at room temperature in 20 mM PBS buffer solution, pH 5.2, under magnetic stirring in Faraday cage. A DC potential of −400 mV was applied; as shown in Figure 2, applying a DC potential of −400 mV allowed the total impedance of the modified electrode to be minimized before the injection of diazinon. The measured impedance spectra were analyzed in terms of electrical equivalent circuits using the analysis program Zview (Scribner Associates, USA). A classical Randles equivalent circuit presented in Figure 3 was used to fit Nyquist plots, including the two resistive elements R_1_ and R_2_, in which the double layer capacitive was replaced by CPE, a constant phase angle element and Warburg impedance Zw (cf. Figure 3). R_1_ corresponds to the Ohmic resistance of the bulk electrolyte and of electrical contacts and R_2_ to the charge transfer resistance between the solution and the modified electrode surface. R_2_ is equal to the diameter of semi-circle. Warburg impedance (Zw) is the specific electrochemical element of diffusion and can be defined as (*Z*_*w*_) = (1 − *j*)^−0.5^, where, σ denotes the Warburg coefficient. CPE takes into account the non-homogeneity of the layer and the impedance of such a non-ideal layer that can be expressed as Z(ω) = CPE^−1^ (jω)^−n^, where ω is a circular frequency and *n* a parameter describing the deviation from an ideal capacitor, varying from 0 to 1 (Katz and Willner, 2003).

## Results and discussion

### Electrochemical characterization of the different steps of biosensor elaboration

#### Cyclic voltammetry characterization (CV)

**Figure 4:**
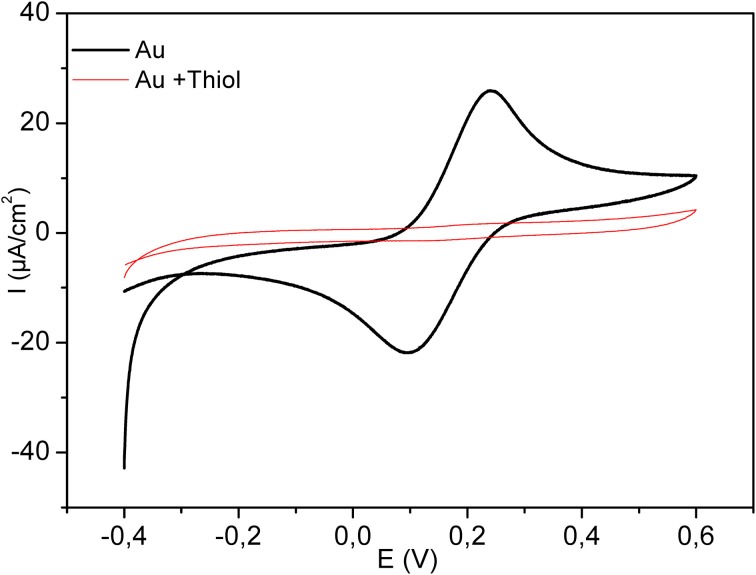
**Cyclic Voltammograms for bare electrode (bold plot) and for modified electrode by thiol (thin line)**. Measurements were performed in 8 mM Fe (CN_6_)^3−/4−^, scan rate 100 mV/s.

Figure 4 shows the electrochemical characterization by CV of the bare and modified electrode in the presence of 8 mM solution of [Fe (CN)_6_]^4−/3^. The potential was swept between −0.4 and 0.6 V, at the scan rate of 100 mV/s. As it is clear in the figure, the complete disappearance of the oxidation and reduction peaks after functionalization with the thiol layer confirms the high insulating proprieties of the dense acid thiol layer.

#### EIS response of the enzymatic biosensor

**Figure 5:**
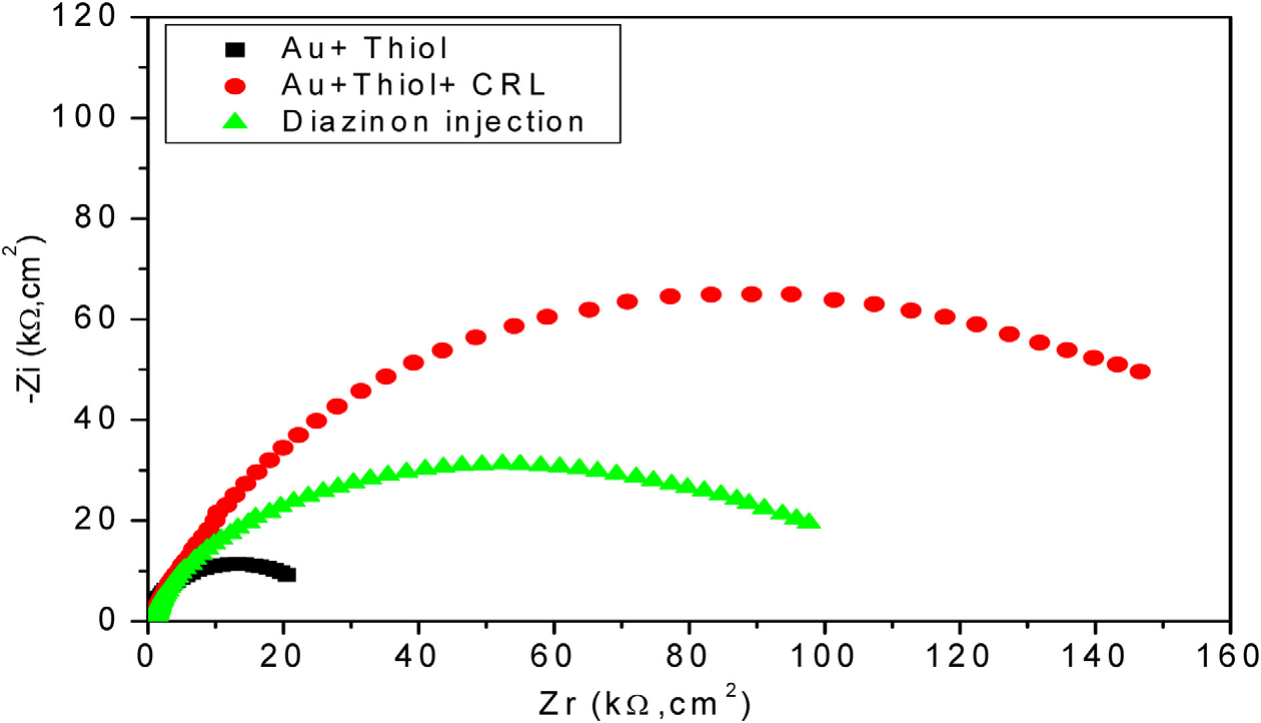
**Nyquist diagrams for different layers of developed biosensor with CRL**. Measurements were carried out in PBS 20 mM, pH 5.2 with a frequency range of 100 mHz–100 kHz.

In Figure 5, a 2.5 fold increase of R_2_ was observed by assembling the first layer on the electrode surface, which basically reflects the insulating proprieties of SAMs (Chen et al., 2005). Contrariwise, a decrease of interfacial impedance, correlated to 1.5 fold decrease of R_2_ was observed following the injection of diazinon, confirming that the hydrolysis of diazinon induces charge redistribution at the functionalized gold electrode/electrolyte interface. The *n*-value was 0.95 for the bare electrode and was reduced to 0.86–0.91 after modification, reflecting only a slight deviation from ideality and a rather capacitive behavior of the corresponding CPE.

### Electrochemical detection of diazinon

#### Variation of impedance after diazinon injections

**Figure 6:**
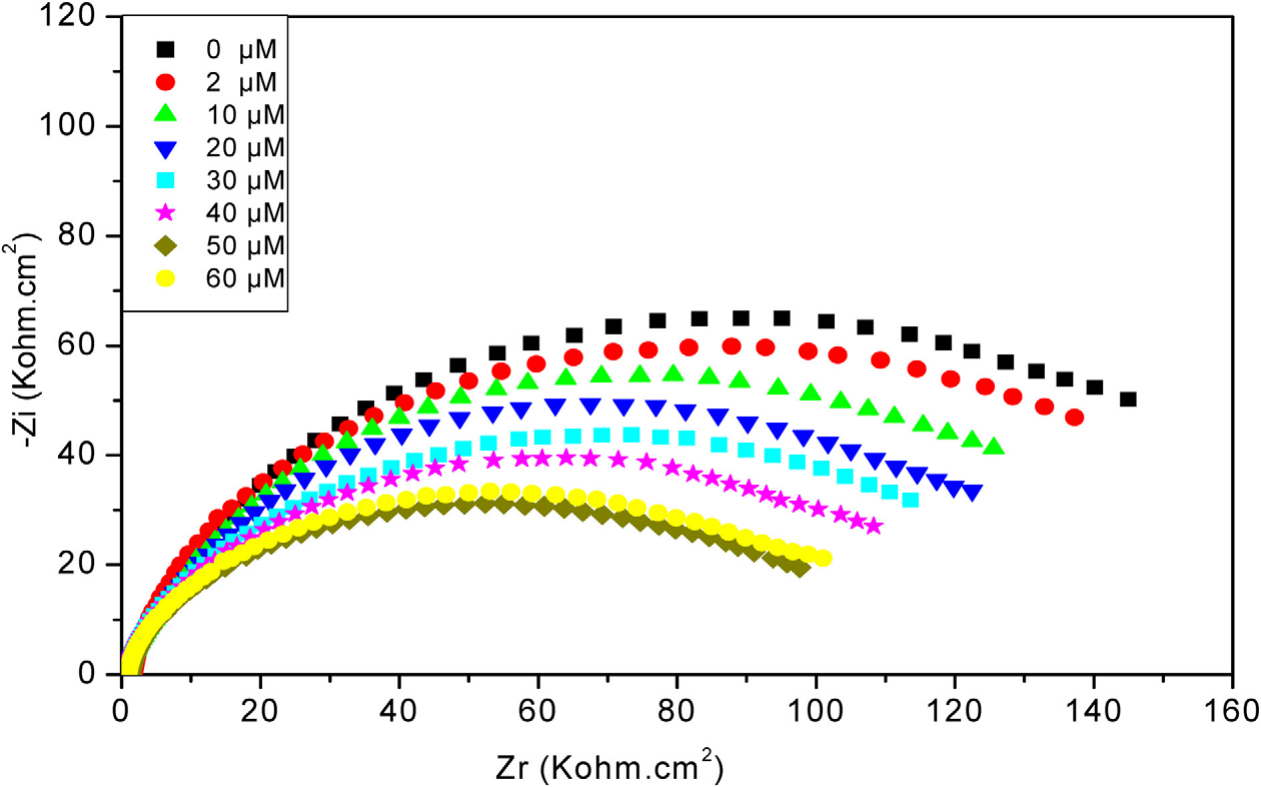
**Nyquist diagrams obtained with CRL biosensor, after injection of different concentrations of diazinon**. Measurements were carried out in PBS 20 mM, pH 5.2 with a frequency range of 100 mHz–100 kHz.

As can be seen in Figure 6, after increasing concentrations of diazinon contact with CRL biosensor, the total impedance decreases, from 2 to 50 μM. For concentrations higher than 50 μM, a saturation effect is observed. The decrease of total impedance is due to a decrease of R_2_, induced by charge redistribution at the functionalized gold electrode/electrolyte interface due to the enzymatic hydrolysis of diazinon, and an associated increase of interface capacitance.

#### Calibration curves for CRL and PPL biosensors

**Figure 7:**
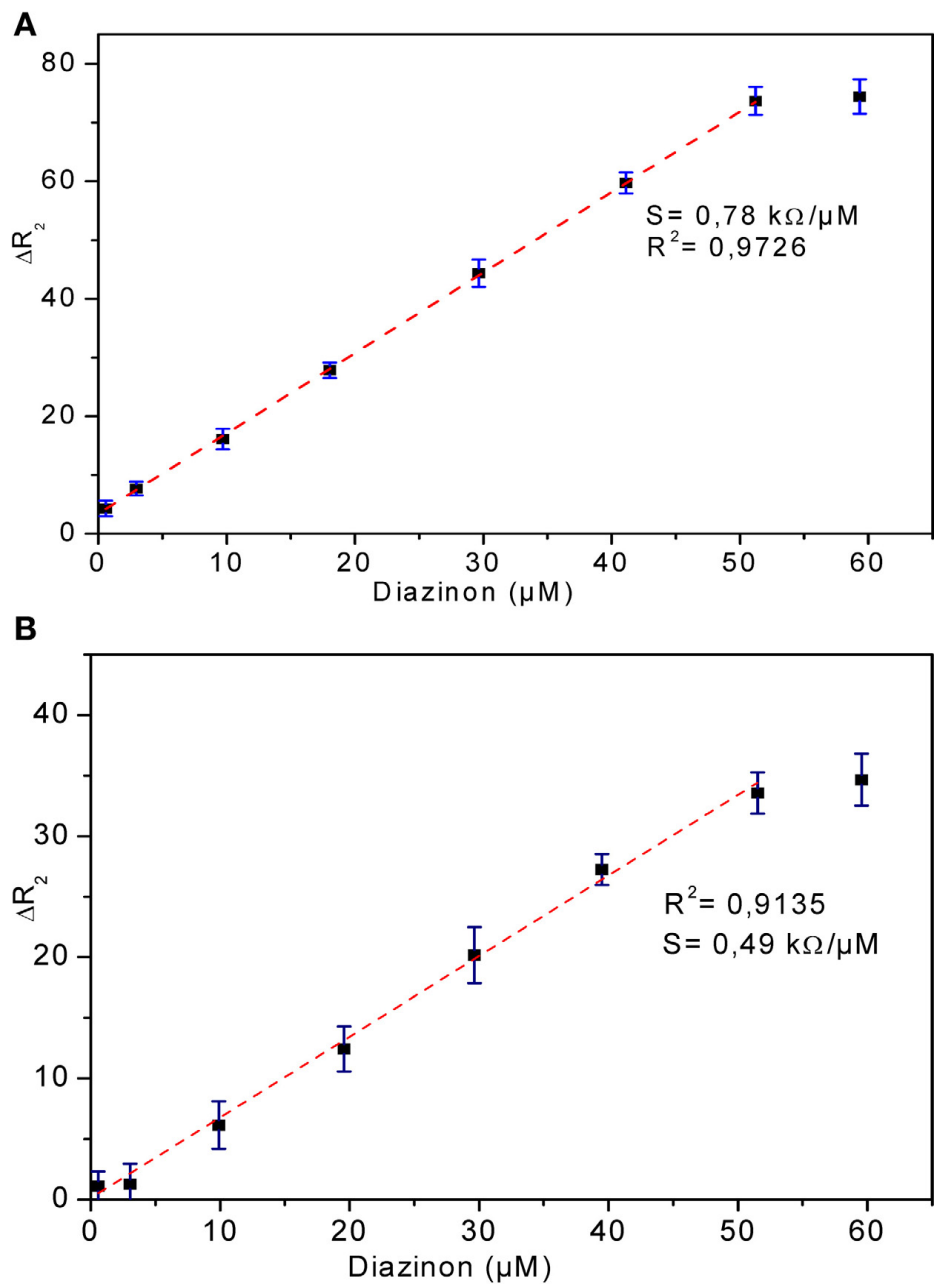
**Calibration curves of diazinon enzymatic biosensors (A) CRL, (B) PPL**. Operational conditions: 20 mM phosphate buffer, pH 5.2 at room temperature. Each point is the mean of three values.

The relationship between biosensor response and diazinon concentration was examined by recording impedance spectra after injection of different concentrations of diazinon in PBS. For both CRL and PPL biosensors, three measurements were performed at each concentration level. To obtain calibration curves, the values of Δ R_2_ = R_2_ − R_2(0)_ were deduced, where R_2(0)_ refers to R_2_ for [diazinon] = 0. Figure 7 presents the calibration plots for both enzymes (a) CRL, (b) PPL, as can be seen, there is a large linear variation of Δ R_2_ with diazinon concentration up to 50 μM for both sensors, with a detection limit of 10 nM for the CRL biosensor and 0.1 μM for the PPL based one. Besides, the relative variation of R_2_ obtained with enzyme CRL is larger (factor 2.5) than that obtained with PPL and as it is shown in Table 1 that the correlation coefficient and sensitivity of CRL biosensor (*S* = 0.78 kΩcm^2^) are better than those obtained by PPL sensor (*S* = 0.49 kΩcm^2^). Therefore, the analytical performances of CRL based biosensor are the best. This CRL based biosensor had a detection limit of 10 nM which is however higher than that biosensors based on inhibition of AchE (Yi et al., 2013), but it presented a large linear range and it was better than enzymatic biosensors already reported like a potentiometric OPH based biosensor (Mulchandani et al., 1999, 2001), an amperometric tyrosinase based biosensor (Tanimoto de Albuquerque and Ferreira, 2007), a tyrosinase based oxygen biosensor (Russell Everett and Rechnitz, 1998), (cf. Table 2). Furthermore, this value is sufficient to allow diazinon determination in industrial waste waters and make the biosensor suitable for on-line environmental monitoring applications.

**Table 1:** **Analytical characteristics of enzymatic biosensors**.

**Enzyme**	**Sensitivity**	**Linear range (μM)**	**Detection limit (μM)**
CRL	0.78 kΩ /μM	Up to 50	0.01
PPL	0.49 kΩ /μM	Up to 50	0.1

**Table 2:** **Analytical characteristics of enzymatic biosensors for the detection of diazinon**.

**Target analyte**	**Transduction**	**Linear range**	**LOD (μM)**	**Enzyme**	**References**
Diazinon	Amperometric	5–50 μM	5	Tyrosinase	Russell Everett and Rechnitz, 1998
Diazinon	Potentiometric	0.13–2.8 mM	5	OPH	Mulchandani et al., 1999
Diazinon	Amperometric	0.06–016 μM	0.06	Tyrosinase	Tanimoto de Albuquerque and Ferreira, 2007
Diazinon	Amperometric	0.46–8.56 mM	2	OPH	Mulchandani et al., 1998
Diazinon	SiQDs fluorescence	/	2.22 × 10^−4^	AchE and ChOx	Yi et al., 2013
Diazinon	Photothermal	/	32.86	AchE	Pogacnik and Franco, 1999
Diazinon	Impedimetric	0.01–50 μM	0.01	CRL	This work
Diazinon	Impedimetric	0.1–50 μM	0.1	PPL	This work

*LOD, limit of detection*.

The enzymatic biosensor was also evaluated for matrix effect of natural compounds in real samples. Diazinon was spiked in water from La Chaudanne river—Lyon (pH of samples water was adjusted from original 7.5 to 5.2 and analyzed within 24 h after collection). the responses of the biosensor in the river water were almost similar to that in the buffer, validating the potential utility of the present biosensor for detection of OPs contaminated natural waters.

#### Selectivity of biosensor

**Figure 8:**
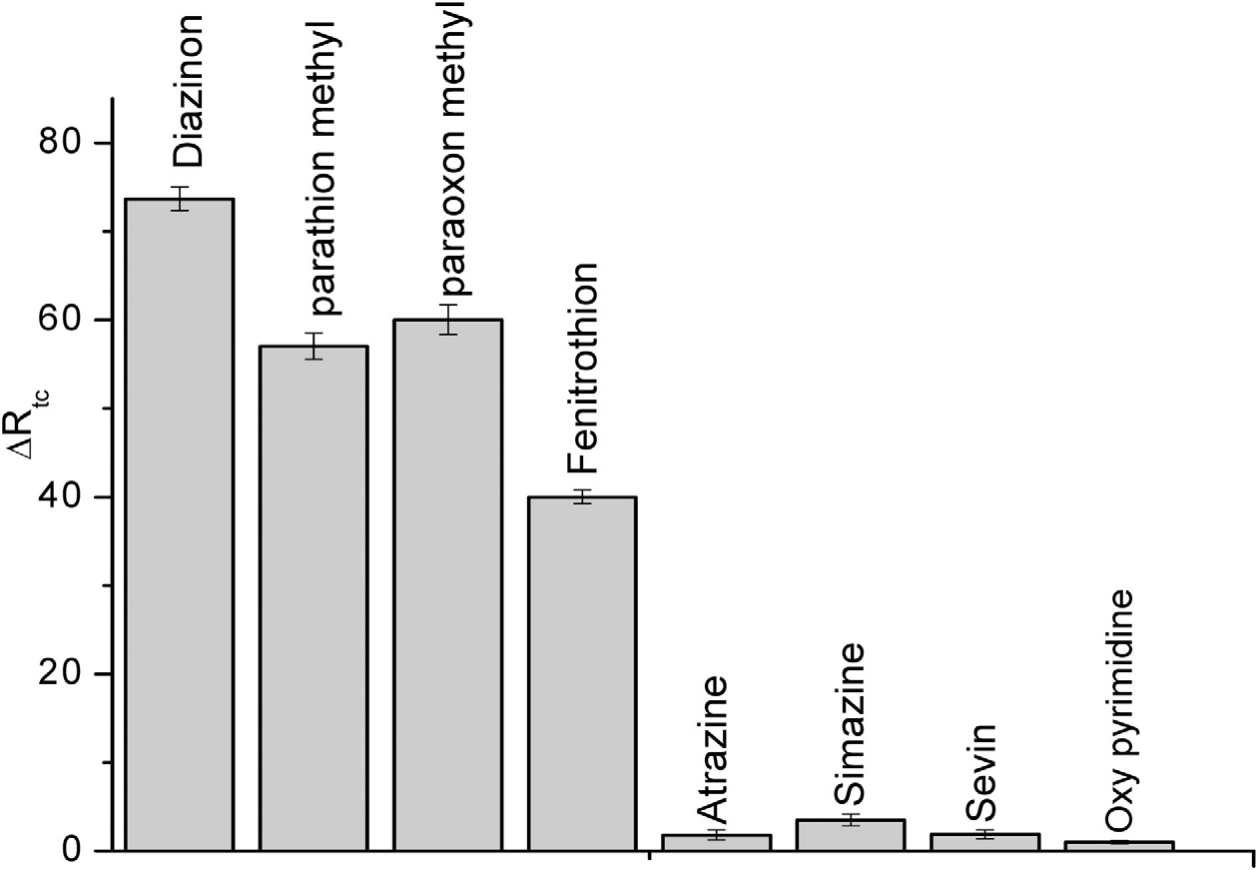
**Interference study using CRL impedimetric biosensor**. Experiments were performed in 20 mM PBS solution, pH 5.2 at room temperature. Each point is the mean of three values.

Selectivity is a fundamental component of biosensor, therefore, it was tested for the detection of some compounds: parathion methyl, paraoxon methyl, fenitrothion, (organophosphate pesticides), atrazine, sevin, simazine (carbamates) and also for oxypyrimidine; the metabolite of diazinon at the concentration of 50 μM, the results are presented in Figure 8. Such as it is clear in the figure, this CRL biosensor is not specific for individual pesticide but to a class of organophosphate pesticides. Conversely, the carbamates and oxypyrimidine did not interfere.

#### Stability and reproducibility of CRL biosensor

Reproducibility and stability are among the key factors of a sensor's performance, the reproducibility of the enzymatic biosensor was tested for three different sensors in the concentration range from 10 to 50 μM. The variation coefficient obtained from three measurements was very good; it was between 2 and 5% in the concentration range studied.

**Figure 9:**
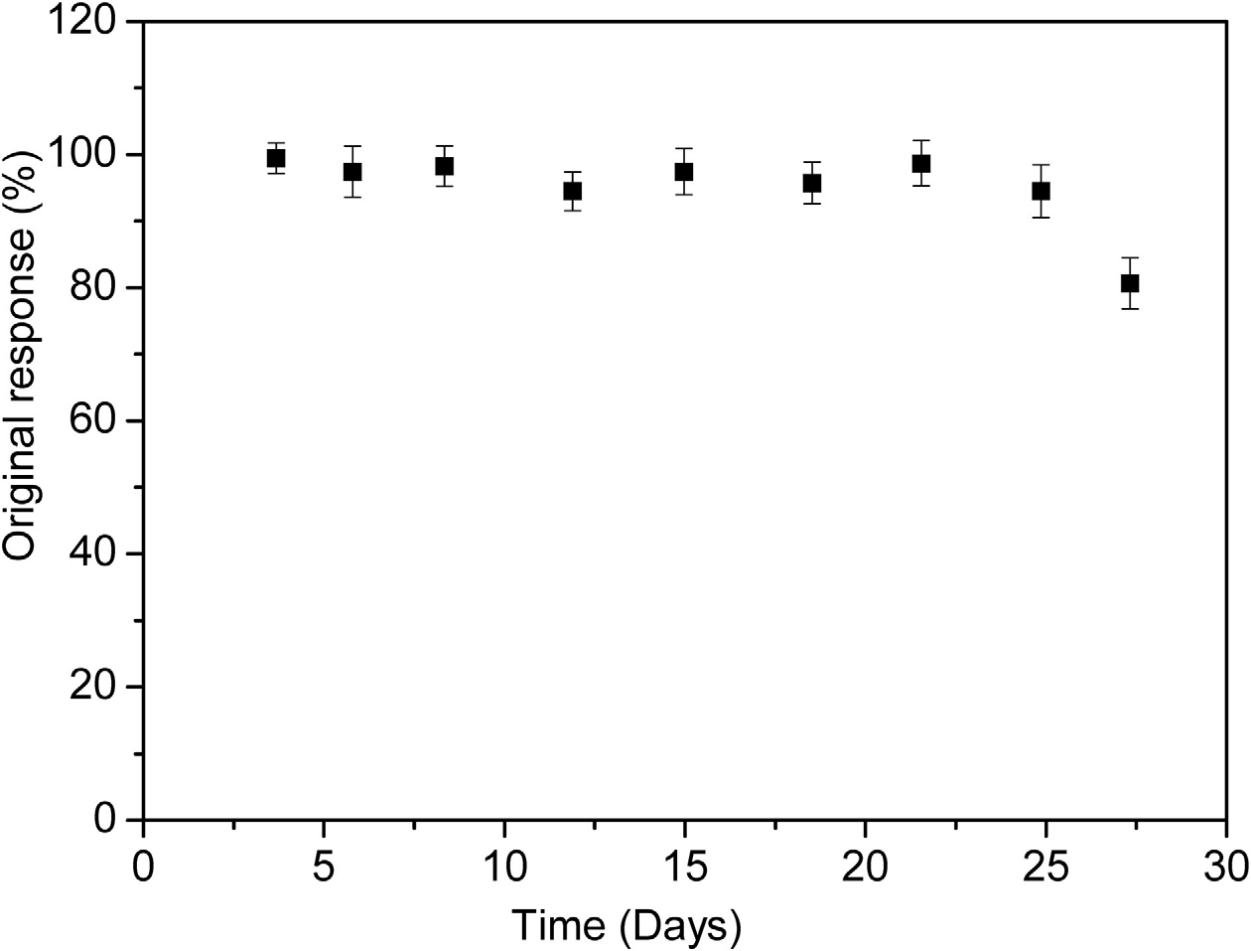
**Stability of enzymatic biosensor, response to 50 μM diazinon in phosphate buffer, pH 5.2**.

To investigate the long-term storage stability and multiple use capability, the enzymatic biosensor was used for 1 month to measure the response to 50 μM of diazinon. During that time, the biosensor was stored at 4°C in 20 mM phosphate buffer (pH 7.2). As demonstrated in Figure 9, the biosensor was stable for 25 days, after this, only 20% of the initial response was lost. Therefore, life time of this biosensor can be estimated to be one month. This storage time was as stable as other previously reports of organophosphate pesticides detection (32 and 30 days) (Mulchandani et al., 2001; Kumar et al., 2006). However, it exceeds those earlier reported in the literature as amperometric screen printed tyrosinase-modified electrodes (10 days) and microbial Sphingomonas sp biosensor for methyl parathion detection (18 days) (Kumar et al., 2006; Tanimoto de Albuquerque and Ferreira, 2007).

## Conclusion

In this work, two impedimetric biosensors were developed for the detection of diazinon. Two different types of lipase were immobilized on the surface of functionalized gold electrode. Both biosensors presented a wide linear range and low detection limits, but the performance of CRL based biosensor is better than PPL biosensor (sensitivity and detection limit). This novel CRL biosensor for direct determination of diazinon is simple, one step with rapid response and large dynamic range. Moreover, it is low cost and does not require any expensive measurement apparatus. Unlike the other biosensors based on OPH or AchE, it is specific only for organophosphate pesticides which make it very promising analytical tool for the detection of organophosphate pesticides in real samples. Thus, it will be ideal for on line monitoring of detoxification processes for the treatment of wastewaters generated by the industrial production of organophosphate-pesticides.

### Conflict of interest statement

The authors declare that the research was conducted in the absence of any commercial or financial relationships that could be construed as a potential conflict of interest.
